# The Nutritional Quality of Commercially Bred Yellow Mealworm (*Tenebrio molitor*) Compared to European Union Nutrition Claims

**DOI:** 10.3390/insects15100769

**Published:** 2024-10-05

**Authors:** Isabelle Noyens, Meggie Van Peer, Sarah Goossens, Carmen Ter Heide, Sabine Van Miert

**Affiliations:** Centre of Expertise Sustainable Biomass and Chemistry, Thomas More University of Applied Sciences, Kleinhoefstraat 4, 2240 Geel, Belgium; meggie.vanpeer@thomasmore.be (M.V.P.); sarah.goossens@thomasmore.be (S.G.); carmen.terheide@thomasmore.be (C.T.H.); sabine.vanmiert@thomasmore.be (S.V.M.)

**Keywords:** yellow mealworm, commercial insect production, protein, fat, nutritional profile, novel food

## Abstract

**Simple Summary:**

In the search for sustainable protein sources, the yellow mealworm (*Tenebrio molitor*) is often regarded as a promising food source. Our research evaluates the applicability of European nutrition claims (Regulation (EU) No. 1924/2006) to commercially bred yellow mealworms, using samples from four Belgian breeders. We analyzed their proximate compositions, fatty acid profiles, and mineral contents to validate these claims. This study improves our understanding of compositional variations among yellow mealworms from different breeders and explores their potential to meet specific European nutritional standards.

**Abstract:**

Due to its potential as a sustainable protein source, the industrial relevance of *Tenebrio molitor*, known as yellow mealworm, is set to increase substantially. Given the novelty of its application in the food industry, knowledge is lacking regarding the nutritional quality of commercially farmed mealworms. This study investigated the nutritional composition of larvae from four different rearing facilities in Belgium and specifically investigated whether their nutritional profiles adhered to defined European nutrition claims (Regulation (EC) No. 1924/2006). In particular, the European nutrition claims “high in protein”, “high unsaturated fat”, “high in fibre” and “rich in P, Mg, K, Zn and Mn” were applicable for all mealworm samples on a dry matter basis. On a fresh matter basis, yellow mealworms were found to be “high in protein”, “high unsaturated fat” and “low in sugar”.

## 1. Introduction

In recent years, insects have emerged as a sustainable, alternative protein source [1,2,3,4] gaining increased attention from scientists, entrepreneurs and governments worldwide. Insects, therefore, will most likely play a significant role in a future, more circular society. However, the application of insects in food systems remains relatively new and, consequently, still is in its formative stages of development. Numerous research questions regarding production and processing optimization must be tackled to fully harness the potential of insects in food applications [5].

The yellow mealworm, *Tenebrio molitor* (Tenebrionidae), is considered one of the most promising insect species for human nutrition. *T. molitor* is a cosmopolitan distributed insect species, originally considered a pest insect due to its natural feeding habits, infesting barns and feeding mostly on stored grain products. They also feed on animal material, vegetables and fruits [6,7]. Nowadays, *T. molitor* is recognized for its ability to convert low-value biomass into valuable nutrients. For this species, novel food dossiers have been evaluated and approved recently in the European Union (EU) for certain applications (Regulation (EC) No. 258/97; Regulation (EU) 2015/2283).

Yellow mealworms have been reported to contain relatively high protein levels, ranging widely from 36% to 60% on a dry matter basis (dmb). Similarly, there is notable variability in the lipid contents of yellow mealworms (6–40% on dmb) [8,9].

Several factors including diet, developmental stage and rearing conditions have been known to influence the nutritional compositions of insects [9,10]. For instance, in a study conducted by Adámková et al. [11], the authors reported that the temperature in which the insects were bred significantly affected the fat content of the larvae. Furthermore, a recent study showed that by feeding mealworms a diet with increased starch, their lipid content doubled and their fatty acid profile changed [12]. Similar results were observed in the study of Krönke and Benning [10]. The authors concluded that a strong effect of dietary carbohydrates and fats on the nutritional composition of the larvae was present. They also observed a weak correlation between dietary protein content and the protein content of the larvae: higher protein contents in diets were reflected in the larvae [10]. Comparable results have been reported by other studies [11,13,14].

The above indicates that the chemical composition of yellow mealworms might vary across commercial breeders, as they might implement different breeding parameters. Processing companies often buy insects from multiple producers to ensure a sufficient and consistent supply. It is important to understand the impact of these factors on the nutritional value of the yellow mealworm to increase quality, which is essential for their suitability in food applications. To support the nutritional quality of food products, the EU has defined specific nutrition claims and their associated criteria, ensuring that when such claims are made, they meet established standards. However, while insects are commonly described as “protein-rich” in scientific research, the data are often not compared to the nutritional claims outlined in Regulation (EC) No. 1924/2006. A nutrition claim refers to any statement that a food possesses specific beneficial nutritional characteristics based on its energy content or its nutrient composition. Nutrition claims are only permitted if they are included in the Annex of Regulation (EC) No. 1924/2006, as amended by Regulation (EU) No. 1047/2012.

Meeting these nutrition claims can increase the added value of insects for food, consequently boosting the emerging insect sector.

Nowak et al. [15] conducted a review comparing compositional data of *T. molitor* found in the literature with the Codex Alimentarius. The authors concluded that the yellow mealworm could be labeled as “high in protein”, “a source of Zn”, “high in Mg” and a “source of” various vitamins. Nevertheless, the authors emphasized the variability in the published values, thereby limiting the applicability of the labels [15].

Regarding the above described, this study investigated the nutritional composition of commercially bred yellow mealworms from four Belgian breeding facilities in terms of their proximate compositions, fatty acid and mineral profiles. The data were compared to the EU regulatory nutrition claims (Regulation (EC) No. 1924/2006). By doing so, this study aimed to obtain a better understanding of the nutritional composition quality of commercially available yellow mealworms intended for food purposes.

## 2. Materials and Methods

### 2.1. Source of the Samples

Multiple samples of blanched and frozen yellow mealworms were sourced from four different Belgian commercial producers (labeled A to D). These samples were shipped in cooled and isolated boxes to the laboratory and, upon arrival, frozen (−20 °C) for preservation for a maximum of 6 months prior to analysis. Limited information on the insect diets was provided by each breeder. Breeder A fed the mealworms with a diet consisting of wheat bran as the dry feed, supplemented with carrots as a moisture source. Breeder B provided wheat bran supplemented with carrots, as well as raw potatoes, to the larvae. Breeder C’s mealworms were fed with wheat bran and carrots, with additional supplementation of oatmeal every two weeks and commercial insect feed every ten days. Yellow mealworms produced at the site of breeder D were exclusively fed with commercial insect feed, supplemented with carrots as a moisture source. Specific breeding conditions were not closely monitored, as breeders often adapted their practices to suit their individual circumstances and practical feasibility.

### 2.2. Proximate Analysis of the Insects

Insect samples were dried until a constant mass at 60 °C was obtained; subsequently, the samples were homogenized by grinding (Tube Mill 100, IKA-Werke GmbH & Co. KG, Staufen, Germany). Samples were stored in an air-tight container at 4 °C. All analyses were performed in duplicate.

The proximate analysis, mineral profile and fatty acid profile were determined as described in Noyens et al. [12]

Dry matter was determined by drying the sample in an oven at 105 °C for 24 h (UF110, Memmert GmbH & Co. KG, Schwabach, Germany). Dry matter was calculated based on the weight loss of the sample.

Crude ash content (CA) was determined based on weight loss by incineration in a muffle furnace (L9/11/SKM, Nabertherm GmbH, Lilienthal, Germany) at 550 °C for 4 h.

Crude lipid or ether extraction (EE) was performed with petroleum ether (BP 40–60°) using Soxhlet equipment.

Crude protein (CP) was determined by analyzing the nitrogen present in the samples using the Kjeldahl method BN EN ISO 5983-1 (2005). The crude protein content of the larvae was calculated with an N to P factor of 5.33 [16].

To determine the chitin content of the larvae, a modified protocol based on the crude fiber analysis method by Van Soest et al. [17] was used, as described by Noyens et al. [12]

The non-fiber carbohydrate (NFC) content was calculated using the following formula:

%NFC = 100% − (%CP + %chitin + %EE + %CA)

### 2.3. Nutritional Analysis of the Insects

The mineral profile was determined using ICP-OES (Optima 4300 ™ DV ICP-OES, Perkin Elmer, Massachusetts, US). P, Ca, Na, K and Mg were determined as macro minerals and Zn, Cu, Fe and Mn were measured as micro minerals. Calibration curves were prepared using standard solutions prepared from a certified stock solution (Multi Element ICP Standard solution (24 E), ChemLab Analytical bvba, Zedelgem, Belgium). Insect samples were prepared in a nitric acid solution using microwave (Mars 2000, CEM Corporation, Matthews, NC, USA) digestion.

The fatty acid profile was determined using a GC-MS (7820A GC system with 5977 E MSD detector, Agilent Technologies, Santa Clara, CA, USA) and SLB-IL60 Capillary Column (30 m × 0.25 mm, 0.20 µm). Relative quantification was performed by comparing the peak surfaces of the fatty acids within the chromatogram to each other. Peaks were identified using a certified stock solution of FAME mixture (FAME Mix (37C) standard solution, Chem Lab Analytical bvba, Zedelgem, Belgium). Fatty acids were additionally grouped into saturated fatty acids (SFAs), monounsaturated fatty acids (MUFAs) and polyunsaturated fatty acids (PUFAs).

### 2.4. Comparison to EU Nutrition Claims

The average nutritional values of the yellow mealworms were compared to the nutrition claims, as described in the Annex of Regulation (EC) No. 1924/2006. The claims relevant to our research and their criteria are presented in Table 1.

To compare the values of the nutritional components of the samples to the described EU limits for food claims, ratios of these values in the nutritional profile must be calculated according to Regulations No. 1624/2006 (list of nutrition claims) and 1169/2011 (EU law on food information to consumers). To determine the applicability of some nutrition claims, it is necessary to calculate the energy value of the mealworm sample as well. The official formula for the calculation of this energy value is provided in Regulation No. 1169/2011, Annex XIV: Energy value (kcal/g) = 4 × NFC + 4 × CP + 9 × EE + 2 × fiber (chitin).

For each mealworm sample, the energy value was calculated to compare the respective contributions of protein or fiber to this whole energetic value. Subsequently, it was verified whether all samples from the different breeders met the minimum requirements for these nutrition claims.

### 2.5. Statistical Analysis

Statistical data analysis was performed using JMP Pro 17.0 software from SAS (Buckinghamshire, UK). Evaluation was performed using Wilcoxon/Kruskal–Wallis tests with the Dunn method as a post hoc test, applying a *p*-value < 0.05.

## 3. Results and Discussion

### 3.1. Proximate Composition

Table 2 presents the average proximate composition of mealworm samples on a dry matter basis from different breeders. Larvae from Breeders B and D had higher fat contents but lower levels of protein, ash, chitin, and non-fiber carbohydrates compared to samples from Breeders A and C. Despite these significant nutritional differences, all mealworm samples met the EU requirements for the “High Protein” and “High Fibre” claims.

According to EU standards, the “High Protein” claim requires that at least 20% of a food’s energy comes from proteins. The lowest average protein content in the mealworm samples still accounted for 31.19 ± 0.84% of their energy value, confirming that yellow mealworm larvae are a rich protein source for human consumption. Additionally, all samples met the “High Fibre” claim due to their high chitin contents. Chitin, which has been suggested in previous studies to offer prebiotic benefits for humans [18,19,20], contributes significantly to the fiber content, as it is the main fiber component in the insect’s exoskeleton [21]. Chitin is partly digestible by humans thanks to the enzyme chitinase, which is present in human gastric fluids. Recent studies on this matter found out that while chitin is not completely digestible, it is not harmful for the body [22].

However, the nutritional data used to compare to the EU claims were based on a dry matter basis, but insects can also be sold fresh for human consumption, such as in paste form for use in sausages and hamburgers. Therefore, it is important to determine which EU nutritional claims are applicable to fresh mealworms (Table 3).

Interestingly, although larvae from Breeders B and D showed lower protein, ash, chitin, and non-fiber carbohydrate levels on a dm basis compared to those from Breeders A and C, their protein and fiber contents were significantly higher on a fm basis. Additionally, the fat and dry matter contents of larvae from Breeders B and D were also higher than those from Breeders A and C.

The higher dm concentrations in the larvae from Breeders B and D may be linked to their elevated fat contents, as fat remains part of the dry matter after the drying process. These increased fat levels could potentially be due to differences in the mealworm diets across breeders. Previous studies have shown that mealworms fed with high carbohydrate diets have higher fat concentrations. For instance, in the study of Noyens et al. [12], mealworms fed with potato cuttings as a moisture source had 35.5% (dm) fat, while mealworms fed with the control (agar) contained only 18.6% fat. Similarly, in the study of Kröncke and Benning [10], mealworms that were fed with potato flakes had 47.8% (dmb) fat and mealworms fed cassava flour, which is also high in carbohydrates, contained 48.6% (dmb) fat. The effect of dietary carbohydrate on the fat concentration of the larvae has also been shown in another study, where the diet with the highest carbohydrate concentration (starch) led to the highest fat concentration in the larvae of all tested diets [23].

To assess the nutritional quality of fresh yellow mealworm larvae from Belgian breeders for commercial food use, their nutritional profiles were compared to EU claims outlined in Regulation (EC) No. 1924/2006. All samples met the criteria for “High Protein” and “Low Sugar”. However, in contrast to the nutritional data on a DM basis, the larvae (FM) no longer qualified as “High in Fibre” due to the dilution effect caused by their high moisture content. This occurs because the protein percentage is evaluated in relation to its contribution to the product’s energy value, while the fiber content is compared to the overall energy value. A positive aspect of the high moisture content is that the larvae now meet the “Low Sugar” claim. The larvae’s low carbohydrate contents align with carbohydrate contents in yellow mealworms published in previous research. A study by Son et al. [24] found that yellow mealworms contained a carbohydrate content of 11.5%, of which only 30% was soluble sugar. Of this total soluble sugar, fructose was the most abundant sugar [24].

### 3.2. Mineral Profile

The average mineral profile of the mealworm samples is shown in Table 4 for macro elements (P, Mg, K, Ca and Na) and micro elements (Zn, Cu, Fe, Mn). As the concentrations of Cu in the samples were below the detection limit of the instrument, i.e., <5 mg/100 g dm, no results for Cu are presented.

In our study, dried larvae from Breeders A and C had higher concentrations of macro- and micro minerals compared to Breeders B and D. Overall, the larvae were low in calcium. Calcium deficiency in yellow mealworms has previously been described in the literature [12,25,26] and is in accordance with our results. In the study of Jajić et al. [23], larvae fed wheat bran showed a significantly higher mineral content than larvae fed other diets. These larvae also showed the lowest fat concentrations.

According to the comparison of the mineral data in this study to Regulation (EU) No. 1169/2011 and the Annex to Directive 90/496/EEC, yellow mealworms were “High in P, Mg, K, Zn and Mn”, as these mineral contents were at least twice their threshold set in nutrition legislation. Not all mealworm larvae samples from Breeders A and D did meet the threshold for Fe. This indicates that potential breeding conditions, e.g., the diet, could affect the mineral composition of mealworm larvae and that standardization is needed. The effect of insect feed on their mineral contents is described in previous research [12]. Not only the feed’s composition but also its bioavailability could affect the mineral uptake and concentration. Overall, yellow mealworm larvae were found to be a mineral-rich food source for P, Mg, K, Zn and Mn, as these mineral concentrations really exceed their thresholds for the nutrition claim. For the other minerals, more research on the effects of insect feed and breeding conditions on these mineral uptakes is desired, especially for calcium, as this is a much-needed mineral in human food and animal feed [27,28,29].

### 3.3. Fatty Acid Profile

The primary fatty acids found in mealworm oil were C16:0 (palmitic acid), C18:1 (oleic acid), and C18:2 (linoleic acid or omega-6), as shown in Table 5. Mealworms from Breeders B and D generally exhibited lower levels of polyunsaturated fatty acids (PUFAs) and higher levels of monounsaturated fatty acids (MUFAs) compared to those from Breeders A and C. The fatty acid profiles of all samples were evaluated against EU nutrition claims, as outlined in Regulation (EC) No. 1924/2006, and all samples met the criteria for “High Unsaturated Fatty Acids”. The samples did not meet any other nutrition claims on fatty acids. Mealworm larvae from Breeders A and C were found to be a “source of omega-3”, as their concentration of alpha-linolenic acid (C18:3) was at least 3 g/100 g product and 3 g/100 g kcal. The mealworm larvae of Breeders B and D did not meet the threshold for this nutrition claim as their percentage of alpha-linolenic acid was lower than 3 g/100 kcal due to their high overall fat percentage, which induces a high energy value of the product.

The high contents of unsaturated fatty acids in yellow mealworms, previously documented in the literature to be up to 77% of their fat content [30,31,32,33], align with the fatty acid profiles observed in this study. This nutritional claim underscores the potential of mealworm oil as a valuable food source with beneficial effects. Mealworm oil is rich in unsaturated fatty acids including omega-3, which are known to support hearth health and reduce inflammation [34]. Additionally, it contains nutrients like oleic acid (C18:1) and linoleic acid (C18:2), which are important for maintaining healthy skin and overall well-being [35].

The largest standard deviations (fluctuations) of the fatty acid concentrations of mealworm samples from the same breeder were observed in palmitic acid (C16:0), oleic acid (C18:1) and linolenic acid (C18:2). The fatty acid concentrations of mealworm larvae from Breeders A and C had the largest standard deviations. This is comparable with the large standard deviations found in their total fat concentrations. This indicates that although mealworm larvae samples from Breeders A and C had lower fat contents than B and D, the variations between the fat and fatty acid contents of the samples were larger.

We assume that elevated fat concentrations in the larvae could be due to a high dietary carbohydrate concentration, as Breeder B supplemented the insect diet with potatoes. This could reflect the ability of insects to (partly) biosynthesize some fatty acids from carbohydrates. However, some insects, as shown for the black soldier fly, lack the ability to synthesize PUFAs and must acquire them from their substrate [36]. Consequently, diets high in sugar lead to an increased production of short-chain fatty acids (MUFAs) in the larvae by de novo synthesis, altering the overall fatty acid composition. However, when the larvae receive less sugars from their diet, they may rely more on dietary fats to build their own fats through an alternative pathway, resulting in a different fatty acid profile, with more resemblance to the feeding substrate [36,37].

For yellow mealworm, it was shown that they can synthesize palmitic, oleic and stearic acids [38], as well as linoleic and linolenic acids [39,40,41]. We hypothesize that PUFAs present in the diet of the yellow mealworm might be used for growth and metabolic processes, while any surplus amounts of nutrients from the diet will be used for the synthesis of fatty acids that demand less energy, for instance, MUFAs, that are then stored or bioaccumulated in the yellow mealworm body. Detailed information on the insect’s feed and breeding conditions need to be monitored in future research to understand these changes in the mealworm’s nutritional composition.

## 4. Conclusions

This study aimed to evaluate the nutritional profiles of commercially bred mealworms from four different breeders in comparison to EU nutrition claims. Specifically, the proximate composition, fatty acid profile and mineral concentrations of yellow mealworms were analyzed. The goal was to assess the quality of yellow mealworm larvae sourced from various Belgian breeders for commercial food purposes, aligning their nutritional profiles with EU regulations, including Regulation (EC) No. 1924/2006.

All samples of yellow mealworm larvae, based on dry matter, met the nutrition claims for “high protein”, “low saturated fat”, and “high fiber”. On a fresh matter basis, the larvae also qualified as “high protein”, “low saturated fat”, and “low sugar”.

When compared to Regulation (EU) No. 1169/2011 and the Annex to Directive 90/496/EEC, the mineral data indicated that yellow mealworms, on a dry matter basis, were “rich in phosphorus, magnesium, potassium, zinc, and manganese”.

Future research should focus on the impact of commercially used insect feed and breeding conditions to better understand the variability in the nutritional compositions of yellow mealworms. Such studies could help breeders to standardize their practices or tailor insect biomass to specific needs. Additionally, monitoring seasonal variations by sampling over time may offer valuable insights into the stability of breeding conditions and their effects on the nutritional composition of mealworms.

Although significant differences were observed in the nutritional profiles of mealworms from different breeders, all samples consistently met the “high protein” claim. Therefore, yellow mealworm larvae can be regarded as a reliable and important protein source for human nutrition.

## Figures and Tables

**Table 1:** The nutrition claims as described in the Annex of Regulation (EC) No. 1924/2006.

Category	Claim	Criteria
Energy value	Low energy	≤40 kcal (170 kJ)/100 g.
	Energy reduced	Reduced by at least 30%, with an indication of the characteristic(s) which make(s) the food reduced in its total energy value.
	Energy-free	≤4 kcal (17 kJ)/100 g.
Fat content	Low fat	≤3 g/100 g.
	Fat-free	≤0.5 g/100 g.
	Low saturated fat	The sum of saturated fatty acids and trans-fatty acids does not exceed 1.5 g/100 g and the sum of saturated fatty acids and trans-fatty acids must not provide more than 10% of energy.
	Saturated fat-free	The sum of saturated fat and trans-fatty acids does not exceed 0.1 g/100 g.
	High monounsaturated fat	At least 45% of the fatty acids present in the product are derived from monounsaturated fat under the condition that monounsaturated fat provides more than 20% of energy of the product.
	High polyunsaturated fat	At least 45% of the fatty acids present derive from polyunsaturated fat under the condition that polyunsaturated fat provides more than 20% of energy of the product.
	High unsaturated fat	At least 70% of the fatty acids present are derived from unsaturated fat under the condition that unsaturated fat provides more than 20% of energy of the product.
Fatty acid content	Source of omega-3 fatty acids	At least 0.3 g of alpha-linolenic acid per 100 g and per 100 kcal, or at least 40 mg of the sum of eicosapentaenoic acid and docosahexaenoic acid per 100 g and per 100 kcal.
	High omega-3 fatty acids	At least 0.6 g of alpha-linolenic acid per 100 g and per 100 kcal, or at least 80 mg of the sum of eicosapentaenoic acid and docosahexaenoic acid per 100 g and per 100 kcal.
Sugar content	Low sugars	≤5 g of sugars/100 g.
	Sugars-free	≤0.5 g of sugars/100 g.
Fiber content	Source of fiber	≥3 g/100 g or ≥1.5 g/100 kcal.
	High fiber	≥6 g/100 g or ≥3 g/100 kcal.
Protein content	Source of protein	≥12% of the energy value of the food is provided by protein.
	High protein	>20% of the energy value of the food is provided by protein.
Mineral content	Source of [name minerals]	The product contains at least a significant amount as defined in the Annex to Directive 90/496/EEC.
	High [name of minerals]	The product contains at least twice the value of “source of mineral”.

**Table 2:** The analytical results (mean values ± standard deviation) of the global composition of yellow mealworms received from Breeders A-D on a dry matter basis compared to the threshold of their applicable nutrition claims as described in the Annex to Directive 90/496/EC. When all determined values of a nutritional parameter meet a nutrition claim, it is marked in bold.

Component	Breeder A	Breeder B	Breeder C	Breeder D	Nutrition Claim
EE g/100 g DM	23.62 ± 2.76 ^b^	33.91 ± 0.64 ^ab^	22.96 ± 4.35 ^b^	35.65 ± 2.97 ^a^	“Low Fat” ≤ 3
CP g/100 g DM	44.96 ± 1.73 ^ab^	42.93 ± 0.58 ^ab^	47.34 ± 2.50 ^a^	42.94 ± 1.02 ^b^	
**Protein part of energy value (%)**	**37.29 ± 2.33 ^a^**	**31.69 ± 0.34 ^ab^**	**39.70 ± 3.76 ^a^**	**31.19 ± 0.84 ^b^**	**“High Protein” > 20**
CA g/100 g DM	5.90 ± 0.69 ^a^	3.36 ± 0.16 ^b^	5.26 ± 0.41 ^ab^	3.62 ± 0.33 ^b^	-
**Chitin g/100 g DM**	**7.97 ± 0.70 ^a^**	**7.11 ± 0.51 ^ab^**	**7.56 ± 0.72 ^ab^**	**6.49 ± 0.58 ^b^**	**“High Fibre” > 6**
NFC g/100 g DM	17.54 ± 2.46 ^a^	12.69 ± 1.37 ^ab^	16.87 ± 3.43 ^ab^	11.30 ± 4.37 ^b^	“Low Sugar” ≤ 5
Energy kcal/100 g DM	479 ± 15 ^a^	542 ± 2 ^a^	479 ± 23 ^ab^	551 ± 14 ^b^	“Low energy”≤ 40

Note: DM = dry matter; FM = fresh matter; EE = ether extract; CP = crude protein; CA = crude ash; NFCs = non-fiber carbohydrates. a–b: values not connected by the same letter in the same row are significantly different (*p* < 0.05). The protein part of the energy value of the product was calculated to compare to the nutrition claims for proteins as described in the Annex of Regulation (EC) No. 1924/2006.

**Table 3:** The analytical results (mean values ± standard deviation) of the global composition of yellow mealworms on a fresh matter basis delivered by the four breeders compared to the threshold of their applicable nutrition claims as described in the Annex to Directive 90/496/EC. When all determined values of a nutritional parameter meet a nutrition claim, it is marked in bold.

Component	Breeder A	Breeder B	Breeder C	Breeder D	Nutrition Claim
DM g/100 g	26.67 ± 2.34 ^b^	31.02 ± 0.46 ^ab^	26.53 ± 2.41 ^b^	33.64 ± 3.27 ^a^	-
EE g/100 g	6.34 ± 0.36 ^b^	10.52 ± 0.03 ^ab^	6.16 ± 0.45 ^b^	11.93 ± 0.16 ^a^	“Low Fat” ≤ 3
CP g/100 g	11.99 ± 0.32 ^b^	13.31 ± 0.01 ^ab^	12.53 ± 0.25 ^ab^	14.43 ± 0.44 ^a^	
**Protein part of energy value (%)**	**40.45 ± 2.51 ^a^**	**31.69 ± 0.34 ^ab^**	**42.32 ± 5.41 ^a^**	**32.56 ± 1.42 ^b^**	**“High Protein” > 20**
CA g/100 g	1.57 ± 0.07 ^a^	1.04 ± 0.02 ^b^	1.39 ± 0.05 ^ab^	1.21 ± 0.03 ^b^	-
Chitin g/100 g g Fiber per 100 kcal	2.12 ± 0.05 ^a^ 1.67 ± 0.16 ^a^	2.20 ± 0.09 ^a^ 1.31 ± 0.09 ^ab^	2.00 ± 0.04 ^a^ 1.59 ± 0.18 ^a^	2.17 ± 0.04 ^a^ 1.18 ± 0.08 ^b^	“Source of Fibre” > 3 OR ≥ 1.5 g/100 kcal
**NFC g/100 g**	**4.65 ± 0.15 ^a^**	**3.94 ± 0.46 ^a^**	**4.46 ± 0.25 ^a^**	**3.90 ± 0.65 ^a^**	**“Low Sugar” ≤ 5**
Energy kcal/100 g	119 ± 15 ^b^	168 ± 2 ^ab^	118 ± 22 ^b^	177 ± 9^a^	“Low energy” ≤ 40

Note: DM = dry matter; FM = fresh matter; EE = ether extract; CP = crude protein; CA = crude ash; NFCs = non-fiber carbohydrates. a–b: values not connected by the same letter in the same row are significantly different (*p* < 0.05). The protein part of the energy value of the product and the fiber content per energy were calculated for comparison with the nutrition claims for proteins as described in the Annex of Regulation (EC) No. 1924/2006.

**Table 4:** The average mineral concentration (± standard deviation) of yellow mealworms, based on DM, from the four different breeders (A–D) compared to the threshold (significant amounts) of the minerals as described in the Annex to Directive 90/496/EC. Values are marked in bold when all the concentrations are at least twice the value of the threshold (“High in [mineral]”) as described in the nutrition claim.

Mineral	Breeder A mg/100 g dm	Breeder B mg/100 g dm	Breeder C mg/100 g dm	Breeder D mg/100 g dm	Threshold mg/100g dm
**Macro elements**					
**P**	**915.07 ± 75.78 ^a^**	**695.86 ± 3.28 ^ab^**	**910.48 ± 92.73 ^a^**	**649.69 ± 56.11 ^b^**	**105**
**Mg**	**308.63 ± 41.85 ^a^**	**202.53 ± 7.41 ^ab^**	**284.81 ± 54.47 ^a^**	**165.15 ± 27.61 ^b^**	**56.3**
**K**	**995.16 ± 67.55 ^ac^**	**767.22 ± 16.89 ^bc^**	**1008.35± 78.45 ^a^**	**818.82 ± 59.62 ^b^**	**300**
Ca	45.63 ± 7.55 ^b^	33.63 ± 2.66 ^b^	61.89 ± 15.91 ^a^	56.89 ± 16.73 ^ab^	120
Na	152.76 ± 12.31 ^ab^	108.79 ± 1.24 ^a^	170.43 ± 26.37 ^b^	143.48 ± 24.20 ^ab^	-
**Micro elements**					
**Zn**	**11.27 ± 1.26 ^a^**	**8.86 ± 0.22 ^a^**	**10.85 ± 1.28 ^a^**	**10.07 ± 0.88 ^a^**	**1.5**
Fe	2.65 ± 0.87 ^a^	2.57 ± 0.37 ^a^	3.46 ± 0.77 ^a^	2.86 ± 1.70 ^a^	2.1
**Mn**	**2.19 ± 0.19 ^a^**	**1.70 ± 0.10 ^ab^**	**1.84 ± 0.17 ^b^**	**1.56 ± 0.07 ^b^**	**0.3**

Note: a–c values not connected by the same letter in the same row are significantly different (*p* < 0.05).

**Table 5:** The average fatty acid content (± standard deviation) of the yellow mealworms delivered by four different breeders (A–D) compared to the threshold of their applicable nutrition claims as described in the Annex to Directive 90/496/EC. Samples meeting a nutrition claim are shown in bold.

Fatty Acid	Breeder A	Breeder B	Breeder C	Breeder D	Nutrition Claim
C12:0%	0.20 ± 0.02 ^b^	0.32 ± 0.01 ^ab^	0.22 ± 0.03 ^b^	0.33 ± 0.14 ^a^	
C14:0%	2.77 ± 0.21 ^b^	4.08 ± 0.13 ^a^	3.29 ± 0.20 ^a^	3.60 ± 0.49 ^a^	
C15:0%	0.28 ± 0.11 ^a^	0.23 ± 0.01 ^ab^	0.18 ± 0.03 ^ab^	0.17 ± 0.03 ^b^	
C16:0%	18.28 ± 1.20 ^a^	17.66 ± 0.16 ^ab^	16.37 ± 0.99 ^b^	18.34 ± 2.26 ^a^	
C16:1%	1.95 ± 0.23 ^a^	3.81 ± 0.31 ^b^	2.68 ± 0.50 ^b^	2.61 ± 0.53 ^ab^	
C16:2%	0.40 ± 0.06 ^ab^	0.40 ± 0.01 ^ab^	0.40 ± 0.08 ^a^	0.31 ± 0.09 ^b^	
C17:0%	0.29 ± 0.03 ^a^	0.24 ± 0.01 ^ab^	0.26 ± 0.05 ^ab^	0.20 ± 0.05 ^b^	
C18:0%	4.03 ± 0.15 ^b^	3.10 ± 0.24 ^b^	4.66 ± 0.34 ^a^	3.93 ± 0.57 ^b^	
C18:1%	32.68 ± 1.81 ^b^	39.14 ± 0.20 ^ab^	37.76 ± 4.43 ^ab^	41.16 ± 4.73 ^a^	
C18:2%	37.27 ± 2.69 ^a^	29.90 ± 0.13 ^ab^	31.85 ± 4.63 ^ab^	27.44 ± 5.67 ^b^	
C18:3%	2.00 ± 0.11 ^a^	1.11 ± 0.03 ^a^	2.14 ± 0.62 ^a^	1.81 ± 0.60 ^a^	“Source omega-3”
C18:3 g/100 g DM	0.47 ± 0.06 ^ab^	0.38 ± 0.02 ^a^	0.47 ± 0.06 ^ab^	0.53 ± 0.07 ^b^	≥0.3 AND
C18:3 g/100 kcal	0.42 ± 0.03 ^ab^	0.20 ± 0.0 ^ac^	0.45 ± 0.14 ^b^	0.27 ± 0.01 ^c^	≥0.3
C20:0%	0.15 ± 0.01 ^a^	0.00 ± 0.00 ^ab^	0.18 ± 0.03 ^b^	0.18 ± 0.03 ^b^	
SFA	25.84 ± 1.35 ^a^	25.64 ± 0.74 ^ab^	25.16 ± 1.29 ^a^	26.69 ± 1.90 ^b^	“Low SFA” < 1.5
MUFA	34.62 ± 1.43 ^a^	42.95 ± 0.72 ^ab^	40.44 ± 2.22 ^b^	43.77 ± 2.29 ^b^	“High MUFA” ≥ 45
PUFA	39.54 ± 1.74 ^a^	31.41 ± 0.41 ^ab^	34.40 ± 2.31 ^a^	29.56 ± 2.52 ^b^	“High PUFA” ≥ 45
**UFA**	**74.45 ± 2.25 ^a^**	**74.60 ± 0.82 ^ab^**	**75.10 ± 3.20 ^a^**	**73.53 ± 3.41 ^b^**	**“High UFA” ≥ 70** **AND** **energy part > 20**
**UFA part of energy value of product (%)**	**32.88**	**41.88**	**32.09**	**41.61**	

Note: SFAs = saturated fatty acids; MUFAs = monounsaturated fatty acids; PUFAs = polyunsaturated fatty acids; UFAs = unsaturated fatty acids. a–c, values not connected by the same letter in the row are significantly different (*p* < 0.05). The calculations for C18:3 fatty acid and UFA to compare them to the nutrition claims for fatty acids were performed as described in the Annex of Regulation (EC) No. 1924/2006.

## Data Availability

Original data are available from the author on request.

## References

[1] Bessa L.W., Pieterse E., Sigge G., Hoffman L.C. (2020). Insects as Human Food; from Farm to Fork. J. Sci. Food Agric..

[2] Bukkens S.G.F., Paoletti M.G. (2005). Insects in the Human Diet: Nutritional Aspects. Ecological Implications of Minilivestock; Role of Rodents, Frogs, Snails, and Insects for Sustainable Development.

[3] Bychkova E., Rozhdestvenskaya L., Podgorbunskikh E., Kudachyova P. (2023). The Problems and Prospects of Developing Food Products from High-Protein Raw Materials. Food Biosci..

[4] Van Huis A., Van Itterbeeck J., Klunder H., Mertens E., Halloran A., Muir G., Vantomme P. (2013). Edible Insects. Future Prospects for Food and Feed Security.

[5] IPIFF (2023). IPIFF Perspectives on the Evolution of the European Insect Sector towards 2030: Current EU Regulatory Status, Existing Opportunities and Prospects for Development.

[6] Ribeiro N., Abelho M., Costa R. (2018). A Review of the Scientific Literature for Optimal Conditions for Mass Rearing *Tenebrio molitor* (Coleoptera: Tenebrionidae). J. Entomol. Sci..

[7] Robinson W.H. (2005). Urban Insects and Arachnids—A Handbook of Urban Entomology.

[8] Osimani A., Garofalo C., Milanović V., Taccari M., Cardinali F., Aquilanti L., Pasquini M., Mozzon M., Raffaelli N., Ruschioni S. (2017). Insight into the Proximate Composition and Microbial Diversity of Edible Insects Marketed in the European Union. Eur. Food Res. Technol..

[9] Van Peer M., Frooninckx L., Coudron C., Berrens S., Álvarez C., Deruytter D., Verheyen G., Van Miert S. (2021). Valorisation Potential of Using Organic Side Streams as Feed. Insects.

[10] Kröncke N., Benning R. (2023). Influence of Dietary Protein Content on the Nutritional Composition of Mealworm Larvae (*Tenebrio molitor* L.). Insects.

[11] Adámková A., Mlček J., Adámek M., Borkovcová M., Bednářová M., Hlobilová V., Knížková I., Juríková T. (2020). *Tenebrio molitor* (Coleoptera: Tenebrionidae)-Optimization of Rearing Conditions to Obtain Desired Nutritional Values. J. Insect Sci..

[12] Noyens I., Schoeters F., Van Peer M., Berrens S., Goossens S., Van Miert S. (2023). The Nutritional Profile, Mineral Content and Heavy Metal Uptake of Yellow Mealworm Reared with Supplementation of Agricultural Sidestreams. Sci. Rep..

[13] Fondevila G., Fondevila M. (2023). Productive Performance of *Tenebrio molitor* Larvae in Response to the Protein Level in the Substrate. J. Insects Food Feed.

[14] Oonincx D.G.A.B., Van Broekhoven S., Van Huis A., Van Loon J.J.A. (2015). Feed Conversion, Survival and Development, and Composition of Four Insect Species on Diets Composed of Food by-Products. PLoS ONE.

[15] Nowak V., Persijn D., Rittenschober D., Charrondiere U.R. (2016). Review of Food Composition Data for Edible Insects. Food Chem..

[16] Boulos S., Tännler A., Nyström L. (2020). Nitrogen-to-Protein Conversion Factors for Edible Insects on the Swiss Market: *T. Molitor*, *A. Domesticus*, and *L. Migratoria*. Front. Nutr..

[17] Van Soest P.J., Robertson J.B., Lewis B.A. (1991). Methods for Dietary Fiber, Neutral Detergent Fiber, and Nonstarch Polysaccharides in Relation to Animal Nutrition. J. Dairy Sci..

[18] Kipkoech C. (2023). Beyond Proteins—Edible Insects as a Source of Dietary Fiber. Polysaccharides.

[19] Refael G., Riess H.T., Levi C.S., Magzal F., Tamir S., Koren O., Lesmes U. (2022). Responses of the Human Gut Microbiota to Physiologically Digested Insect Powders or Isolated Chitin Thereof. Future Foods.

[20] Stull V.J., Weir T.L. (2023). Chitin and Omega-3 Fatty Acids in Edible Insects Have Underexplored Benefits for the Gut Microbiome and Human Health. Nat. Food.

[21] Finke M.D. (2007). Estimate of Chitin in Raw Whole Insects. Zoo Biol..

[22] Kim S.Y., Kwak K.W., Park J.Y., Park E.S., Nam C.J., An K.S., Kim H.J., Yoon H.J., Kim Y.S., Park K. (2023). Evaluation of Subchronic Oral Dose Toxicity and Allergen of Freeze-Dried Powder of *Locusta migratoria* (Orthoptera: Acrididae) as a Novel Food Source. Toxicol. Res..

[23] Jajić I., Krstović S., Petrović M., Urošević M., Glamočić D., Samardžić M., Popović A., Guljaš D. (2022). Changes in the Chemical Composition of the Yellow Mealworm (*Tenebrio molitor* L.) Reared on Different Feedstuffs. J. Anim. Feed Sci..

[24] Son Y.J., Hwang I.K., Nho C.W., Kim S.M., Kim S.H. (2021). Determination of Carbohydrate Composition in Mealworm (*Tenebrio molitor* L.) Larvae and Characterization of Mealworm Chitin and Chitosan. Foods.

[25] Selaledi L., Mbajiorgu C.A., Mabelebele M. (2020). The Use of Yellow Mealworm (*T. Molitor*) as Alternative Source of Protein in Poultry Diets: A Review. Trop. Anim. Health Prod..

[26] Zwart P., Rulkens R.J. (1979). Improving the Calcium Content of Mealworms. Int. Zoo Yearb..

[27] González-Vega J.C., Stein H.H. (2016). Digestibility of Calcium in Feed Ingredients and Requirements of Digestible Calcium for Growing Pigs. Anim. Prod. Sci..

[28] Kwiatkowska K., Winiarska-Mieczan A., Kwiecień M. (2017). Feed Additives Regulating Calcium Homeostasis in the Bones of Poultry—A Review. Ann. Anim. Sci..

[29] Pu F., Chen N., Xue S. (2016). Calcium Intake, Calcium Homeostasis and Health. Food Sci. Hum. Wellness.

[30] Dreassi E., Cito A., Zanfini A., Materozzi L., Botta M., Francardi V. (2017). Dietary Fatty Acids Influence the Growth and Fatty Acid Composition of the Yellow Mealworm *Tenebrio molitor* (Coleoptera: Tenebrionidae). Lipids.

[31] Finke M.D. (2002). Complete Nutrient Composition of Commercially Raised Invertebrates Used as Food for Insectivores. Zoo Biol..

[32] van Broekhoven S., Oonincx D.G.A.B., van Huis A., van Loon J.J.A. (2015). Growth Performance and Feed Conversion Efficiency of Three Edible Mealworm Species (Coleoptera: Tenebrionidae) on Diets Composed of Organic by-Products. J. Insect Physiol..

[33] Bogusz R., Bryś J., Onopiuk A., Pobiega K., Tomczak A., Kowalczewski P.Ł., Rybak K., Nowacka M. (2024). The Impact of Drying Methods on the Quality of Blanched Yellow Mealworm (*Tenebrio molitor* L.) Larvae. Molecules.

[34] Jankauskienė A., Aleknavičius D., Andrulevičiūtė V., Mockus E., Bartkienė E., Juknienė I., Kiseliovienė S., Zavistanavičiūtė P., Zaborskienė G., Kabašinskienė A. (2024). Nutritional Composition and Safety Parameters of Mealworms (*Tenebrio molitor*) Reared on Substrates Derived from By-Products. Appl. Sci..

[35] Verheyen G.R., Meersman F., Noyens I., Goossens S., Van Miert S. (2023). The Application of Mealworm (*Tenebrio molitor*) Oil in Cosmetic Formulations. Eur. J. Lipid Sci. Technol..

[36] Hoc B., Genva M., Fauconnier M.L., Lognay G., Francis F., Caparros Megido R. (2020). About Lipid Metabolism in *Hermetia illucens* (L. 1758): On the Origin of Fatty Acids in Prepupae. Sci. Rep..

[37] Thompson S.N. (1979). The Effects of Dietary Carbohydrate on Larval Development and Lipogenesis in the Parasite, *Exeristes roborator* (Fabricius) (Hymenoptera: Ichneumonidae). J. Parasitol..

[38] Canavoso L.E., Jouni Z.E., Karnas K.J., Pennington J.E., Wells M.A. (2001). Fat Metabolism in Insects. Annu. Rev. Nutr..

[39] Fasel N.J., Mene-Saffrane L., Ruczynski I., Komar E., Christe P. (2017). Diet Induced Modifications of Fatty-Acid Composition in Mealworm Larvae (*Tenebrio molitor*). J. Food Res..

[40] Mattioli S., Paci G., Fratini F., Dal Bosco A., Tuccinardi T., Mancini S. (2021). Former Foodstuff in Mealworm Farming: Effects on Fatty Acids Profile, Lipid Metabolism and Antioxidant Molecules. LWT.

[41] Sánchez-Muros M.J., Barroso F.G., Manzano-Agugliaro F. (2014). Insect Meal as Renewable Source of Food for Animal Feeding: A Review. J. Clean. Prod..

